# Cystitis: significant associations between pathology, histology, and quantitative bacteriology in sows, a cross-sectional study

**DOI:** 10.1186/s40813-023-00336-8

**Published:** 2023-09-26

**Authors:** Lola Kathe Tolstrup, Páll Skúli Leifsson, Luca Guardabassi, Jens Peter Nielsen, Ken Steen Pedersen

**Affiliations:** 1 Department of Veterinary and Animal Sciences, Faculty of Health and Medical Sciences, University of Copenhagen, Groennegaardsvej 15, 1870 Frederiksberg C, Denmark; 2 Ø-Vet A/S, Koeberupvej 33, 4700 Naestved, Denmark

**Keywords:** Bacteriuria, Cystitis, *E. coli*, Histopathology, Pathology, Porcine, Sows, Cystitis, Urinary tract infections

## Abstract

**Background:**

The importance of cystitis in pig production is controversial and sparse information is available on its frequency and etiology in sows. The aim of this study was to determine the prevalence of bacteriuria, macroscopical and histological lesions of the urinary bladder in culled sows. Urinary bladders and urine samples were obtained from 176 culled sows at slaughter. The urine samples collected by cystocentesis were analyzed to determine bacterial content and pathological findings, macroscopic as well as microscopic, of the bladder were recorded for each sow.

**Results:**

The prevalence of bacteriuria, defined by bacterial concentrations ≥ 10^3^ colony forming units per mL of urine, was 34%. *Escherichia coli* was isolated from 69% of the samples with bacteriuria. Redness of the mucosa was the most frequently observed macroscopic change of the bladder. Intense redness and presence of pus was considered significant pathological changes and occurred in 27% of the urinary bladders. The histopathological examination showed that mononuclear cells were the predominant type of cell infiltration in the bladder mucosa, while neutrophils occurred in very few samples. The criteria for cystitis determined by histopathology were met in 46% of the samples. The criteria were based on presence of hyperemia, edema, and inflammatory cell reaction defined as 40 or more neutrophils or mononuclear cells per high power field. All three indicators of cystitis were significantly associated with each other (p < 0.05) at sow level.

**Conclusion:**

This study shows that signs of cystitis are common in culled sows. The prevalence of cystitis was 34% based on bacteriological examination, 27% based on macroscopic examination and 46% based on histological examination. Significant associations were found between the three indicators of cystitis: bacteriuria, pathological and histological lesions of the bladder. Based on macroscopic changes and histopathology of the bladder, the cut-off of ≥ 10^3^ colony forming units per mL of urine seems to be appropriate for assessing urine cultures obtained by cystocentesis.

## Background

Cystitis is due to infection of the urinary bladder and can lead into severe upper urinary tract infections (UTI), such as pyelonephritis [1, 2]. In sows cystitis has been associated with uterine infections, such as MMA (metritis, mastitis and agalactia) [3] and poor reproductive performance. While the clinical importance of cystitis in sows is controversial, pyelonephritis is considered a severe disease [1, 4] and can cause death [4, 5]. Cystitis and UTI have been associated with low farrowing rate [6], decreased conception rate [7–9], smaller litter sizes [10] and increased risk of abortion [9]. However, one study could not confirm that urinary tract infection (UTI) and cystitis have a negative impact on reproduction in sow herds [11].

Diagnosis of cystitis has previous been reviewed [12]. The definition of cystitis in sows has not been standardized. Various criteria, including bacteriuria [13–17], demonstration of nitrite and/or blood using reagent strip tests [18], macroscopic pathology [13, 15, 16, 19–22], histopathology [13, 16, 17, 20, 23] and ultrasound examination have been used as indicators of cystitis/UTI. A resent review suggested that a diagnostic workup may include an initial ultrasound examination of the urinary bladder that is followed by collecting a urine sample for urine analysis [24]. Earlier studies have reported a variable prevalence of cystitis ranging between 27 and 53% based on histopathology [13, 16, 20, 23], between of 12.2 and 68% based on macroscopic pathological changes [13, 15, 21, 22], and between 23 and 38% based on bacteriuria [13, 17, 20]. In the studies conducted during the last 20 years *E. coli* was the predominant species isolated from sow urine [13, 15, 17, 21, 23, 25]. Some earlier studies also isolated *Actinobaculum suis.* [27–29]. Most of the studies investigating cystitis and UTI in sows are relatively old, and there is therefore sparse updated knowledge concerning prevalence of histological cystitis lesions, macroscopic bladder pathology, bacteriuria and the bacterial species involved. To be able to assess the importance of cystitis in pig production, information about the frequency of this disease condition is essential.

The aim of this study was to determine the prevalence of bacteriuria, macroscopical and histological lesions of the bladder in culled sows. The validity of each of these indicators was evaluated for post-mortem diagnosis of cystitis.

## Results

### Herd data

The study included 176 sows originating from 143 different herds. Herd details were only available for 113 herds since 30 sows had lost the ear-tag in the slaughter process. Geographically the herds in the study represented a large part of Denmark, distributed as 84 (74%) from western Denmark (Jutland) and 29 (26%) from eastern Denmark, including Zealand and Funen.

The average herd size was 731 sows, with a standard deviation of 361 sows, a minimum of 100 sows and a maximum of 1630 sows. Three different production herd types were registered: sow herds producing piglets and/or slaughter pigs (n = 101, 89%), breeding herds which produce gilts 84 (74%), and herds with free range/organic sows (n = 2, 2%). The total is above 100% as some farms had more than one production type.

### Bacteriological examination

**Fig. 1:**
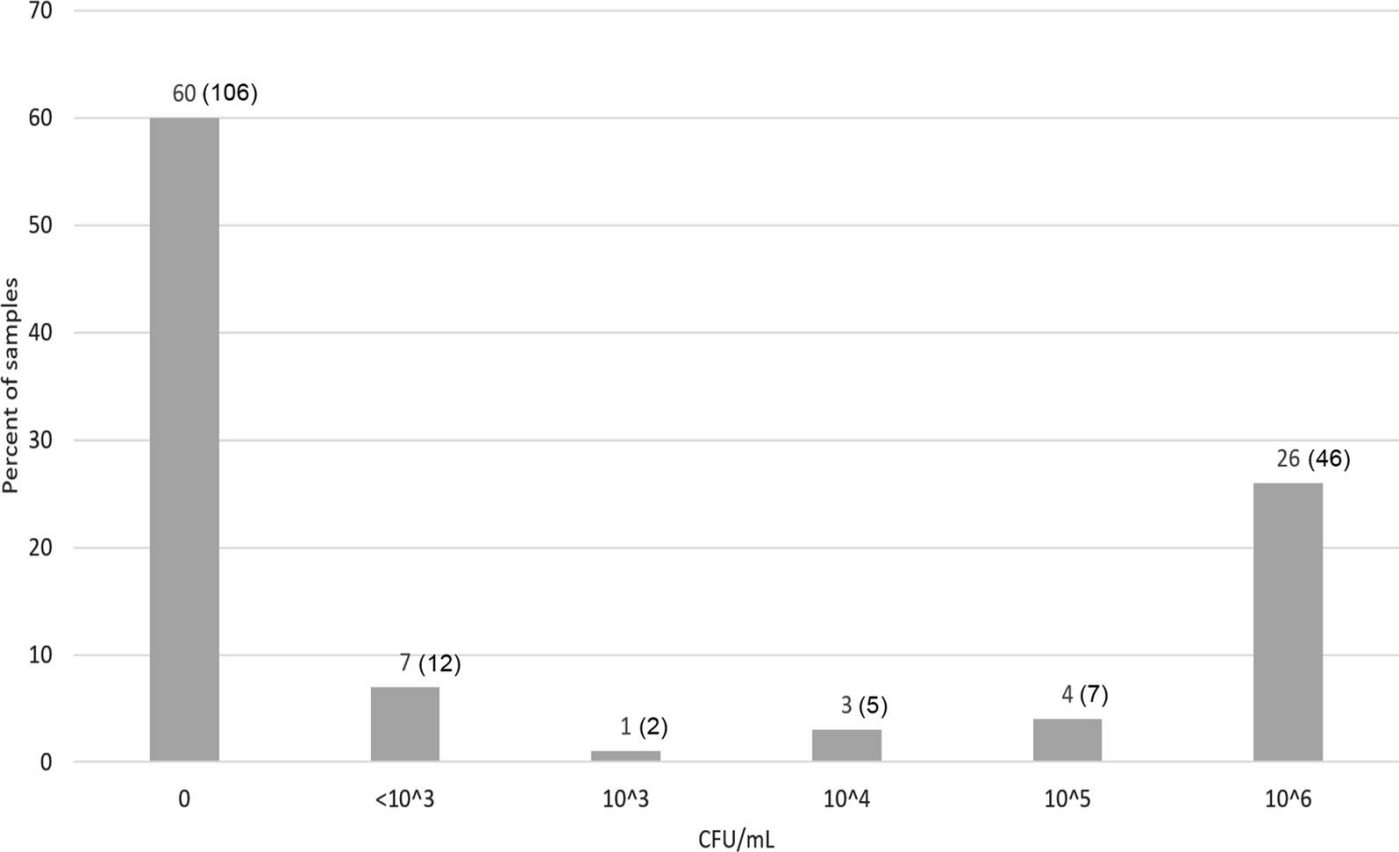
Colony forming units per ml urine in 176 urine samples from culled sows. Numbers are displayed as percent of all 176 urine samples. Number of samples for each category are provided in parentheses

In total 71 (40%) out of the 176 urine samples showed bacterial growth with a minimum of one colony per plate. Bacteriuria with ≥ 10^3^ CFU/mL was demonstrated in 59 (34%) of the samples. The distribution of samples according to CFU categories is shown in Fig. 1.

The distribution of bacteria isolated from all samples is displayed in Table 1. All samples except one resulted in pure cultures. The only mixed culture contained *Streptococcus pluranimalium* and *Pasteurella mairii*.

**Table 1:** Bacterial detected from 71 sow urine samples with bacterial growth

Bacterial species	Number (%) of positive samples < 10^3^ CFU/ml	Number (%) of positive samples ≥ 10^3^ CFU/mL
*Escherichia coli*	2 (2.8)	41 (57.7)
*Streptococcus pluranimalium* and *Pasteurella mairii*	0	1 (1.5)
*Staphylococcus spp*	3 (4.2)	7 (9.9)
*Streptococcus spp.*	1 (1.5)	2 (2.8)
*Enterococcus spp.*	0	2 (2.8)
*Bacillus sphaericus*	1 (1.5)	0
*Pasteurella mairii*	0	1 (1.5)
*Micrococcus luteus*	1 (1.5)	0
*Klebsiella pneumoniae*	0	1 (1.5)
*Acinetobacter* sp.	1 (1.5)	0
Isolates not identified	3 (4.2)	4 (5.6)

The following genera were isolated from sows with bacteriuria (≥ 10^3^ CFU/mL): *Escherichia, Streptococcus, Staphylococcus, Enterococcus, Pasteurella* and *Klebsiella*. *Bacillus, Micrococcus* and *Acinetobacter* were only isolated from samples that were considered as contaminated (< 10^3^ CFU/mL). Moreover, 95% (41/43) of all *E. coli*-positive samples were isolated from sows with bacteriuria, whereas this was the case for 70% (7/10) of the *Staphylococcus*-positive samples. The *Staphylococcus* species identified were *S. equorum, S. haemolyticus, S. vitulinus, S. saprophyticus, S. simulans, S. hyicus* and *S. aureus.* One *Staphylococcus* species could not be identified. The *Streptococcus* species identified were *S. suis, S. dysgalactiae* and *S. gallolyticus.* The *Enterococcus* species identified was *E. faecalis*. One *Enterococcus* isolate could not be identified.

### Macroscopic bladder pathology

Significant acute pathological changes defined by intense redness (dark pink to red) or mild redness (light or pale pink) with presence of pus were observed in bladders from 48 (27%) sows. Additionally, chronic changes (thickening of mucosa) were seen in 3 (2%) samples with intense redness.

Abnormal content (pus, blood and concrements) was observed in 65 (37%) of the 176 bladders. Other findings are shown in Table 2.

**Table 2:** Macroscopic pathological findings in bladders from 176 sows

Pathological finding	Number of sows (%)
Abnormal content	
Pus	23 (13%)
Blood	4 (2%)
Concrements	48 (27%)
Mucosal redness	
Mild	73 (41%)
Intense	41 (23%)
Thickening of mucosa	
Present	3 (2%)
Bladder weight (g)	
Mean [min; max] standard deviation	263 [70; 560] 89

No significant association was found between presence of pus and concrements in the urinary bladders (P = 0.39). Intense redness was observed in 13 of 23 bladders (57%) with pus (P < 0.001, OR = 5.7 [2.1; 16.3]). Mild redness only counted 6 of the 23 bladders (26%) with pus (P = 0.1). On the contrary, mild redness was significantly more predominant in samples with concrements (p-value = 0.006, OR = 2.6 [1.2; 5.4]), with an overall prevalence of 58% (28/48). Intense redness was only observed in 25% (12/48) of the samples with concrements (p-value = 0.8).

**Fig. 2:**
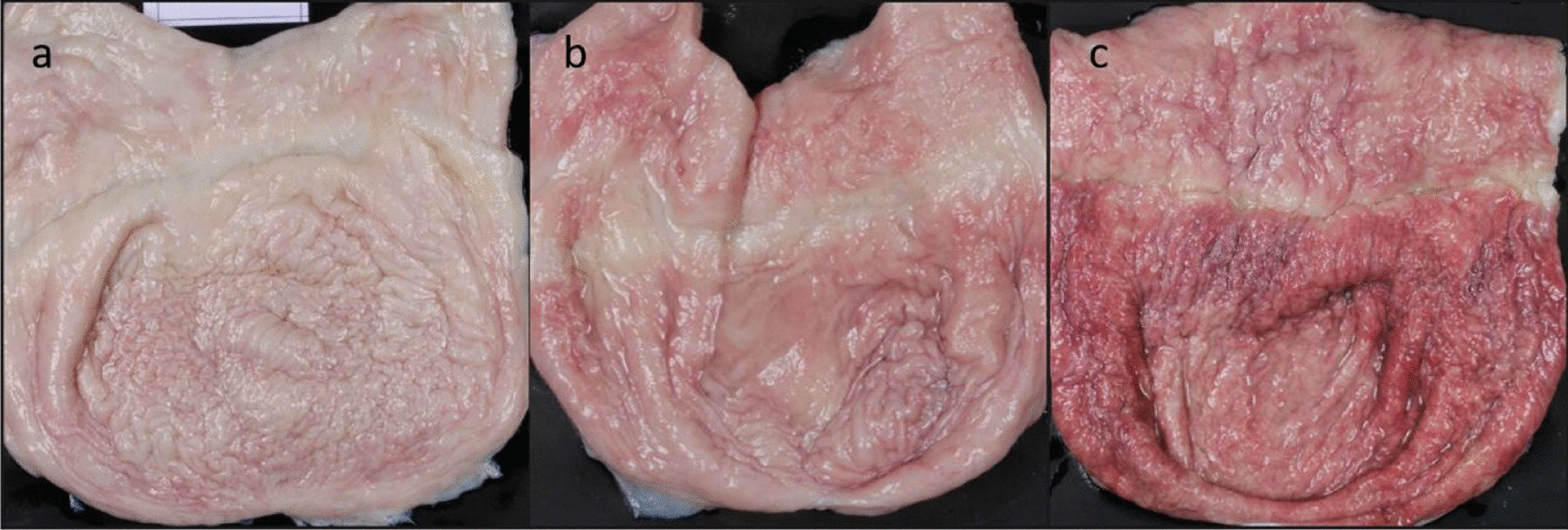
Pictures of different grades of redness in the bladders of culled sows. **a** no redness, **b** mild redness, **c** intense redness. The plastic strip used to close the bladder after removal from the carcass has caused the horizontal lighter line at the top 1/3 of the bladders

Figure 2 displays bladders with different macroscopic pathological changes. Mild redness was the only finding significantly associated with concrements. All comparisons between concrements and other macroscopic pathological changes, bacteriuria and histopathology were non-significant.

The bladder weight was increased by 55 g in bladders containing pus (P = 0.006) and 24 g in the bladders containing concrements (P = 0.05) compared to the bladders without pus and concrements respectively. In bladders with intense redness, the bladders’ weight increased by 83 g (P < 0.001) compared to bladders without redness and 61 g (P = 0.001) compared to bladders with mild redness. No difference was seen between bladders without redness and bladders showing mild redness. Furthermore, it was found that macroscopic intense redness was associated with hyperemia, as determined by histopathology (P = 0.01, OR = 2.9 [1.3; 7.0]), while mild redness was not (P = 0.9).

### Histopathology

**Fig. 3:**
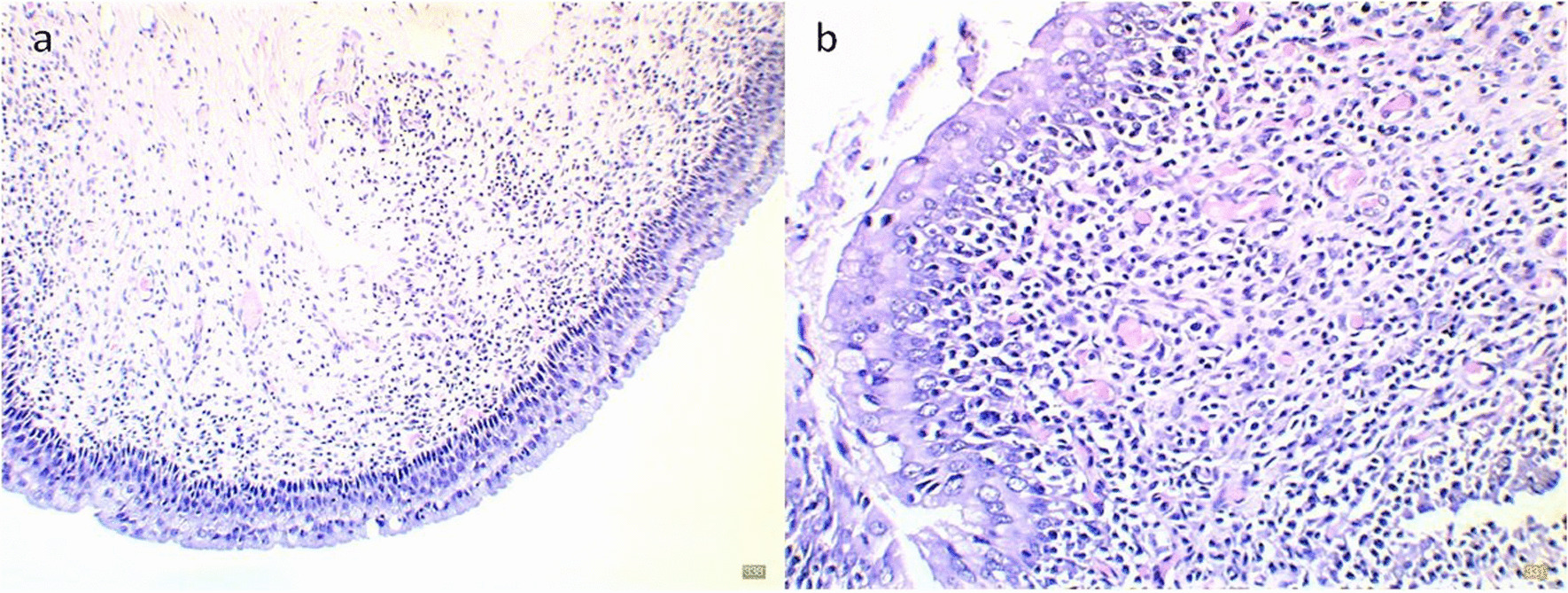
Histological images of chronic cystitis from a culled sow’s urinary bladder. **a** Mononuclear cell infiltration and hyperemia at ×10 objective, **b** Mononuclear cell infiltration and hyperemia at ×40 objective

Lesions indicative of cystitis were observed by histopathology in 81 (46%) of the sows. Four bladders (2%) were categorized with acute cystitis, 56 (32%) with chronic cystitis (Fig. 3) and 21 (12%) with chronic-active cystitis. Lymphocytic foci were seen in 54 bladders (30%) and edema and hyperemia in 58 (34%) and 89 (51%) bladders, respectively. Table 3 shows that the median number of mononuclear cells was 31 cells pr. high-power field (HPF), defined as one field of view in the microscope at 40× magnification, while the median number for neutrophils was 0. Neutrophils were observed in 34 bladders but 26 of those had only 1 pr. HPF.

**Table 3:** Results from histopathological cell counts of samples from 176 bladders

	Min	1st quartile	Median	3rd quartile	Max
Mononuclear cells	4	17	31	98	533
Neutrophils	0	0	0	0	18

Cells pr. high-power field (HPF), defined as one field of view in the microscope at 40× magnification

Edema and hyperemia demonstrated by histology were significantly associated (P < 0.001, OR = 3.9 [1.9; 8.4]). Significantly more mononuclear cells were observed if edema and/or hyperemia were present (all p-values were less than 0.05) and in bladders with lymphocytic foci. Edema was also statistically associated with presence of neutrophils (P = 0.008), whereas no statistical association was found between numbers of neutrophils and presence of lymphocytic foci or hyperemia. A significantly higher number of mononuclear cells were observed if at least 1 neutrophil was present (median = 75 cells), as compared to if no neutrophils were present (median = 28 cells) (P = 0.08).

### Association between bacteriuria, significant macroscopic pathological changes and histological cystitis lesions

The associations between bacteriuria, macroscopical and histopathological findings are shown in Table 4. Acute cystitis was only observed in four samples.

**Table 4:** Associations between different histopathological diagnosis of cystitis, bacteriuria and significant macroscopical pathological changes (n = 176 sows)

		Bacteriuria			Significant pathological changes		
		+	−		+	−	
Histological cystitis lesions in general	+	52	29	p < 0.001 OR = 22.0 [8.8; 64.1]	35	46	p < 0.001 OR = 5.2 [2.4; 12.2]
	−	7	88		12	83	
Acute histological cystitis	+	2	2	p = 0.603 OR = 2.0 [0.1; 28.4]	2	2	p = 0.290 OR = 2.8 [0.2;39.8]
	−	57	115		45	127	
Chronic histological cystitis	+	38	26	p < 0.001 OR = 6.3 [3.0; 13.4]	26	38	p = 0.002 OR = 3.0 [1.4; 6.3]
	−	21	91		21	91	
Chronic active histological cystitis	+	17	4	p < 0.001 OR = 11.3 [3.4; 48.7]	10	11	p = 0.03 OR = 2.9 [1.0; 8.2]
	−	42	113		37	118	

Significant pathological changes was defined as either acute by intense redness or mild redness with presence of pus or as chronic by massive thickening of mucosa.

## Discussion

The aim of this study was to determine the prevalence of bacteriuria, macroscopical and histological lesions of the bladder in culled sows. The validity of each of these indicators was evaluated for post-mortem diagnosis of cystitis.

For this study, information about the parity of the sows and cause of culling is missing. Further, herd data were restricted to those available from the Danish Central Husbandry Register and the Danish SPF-Sus Herd Database. This is a limitation to the study and additional sow and herd information could have given valuable knowledge about possible confounders in relation to the weight of the bladder and the risk of bacteriuria. Therefore, a study where these registrations were included could be relevant.

Another limitation to the study is that because sows may be culled because of reproductive failure or health problems [21], the sample may not be totally representative for live sows in production herds. In addition, many sows are slaughtered just after weaning, meaning that many of the samples possible came from sows placed in the same time of their reproductive cycle. An older study has shown that the prevalence of bacteriuria is similar during a reproductive cycle [11], although information on this area is sparse. Moreover, this older study was conducted at a time where sows were typically fixed during pregnancy, whereas now sows must be loose housed. The influence of housing on the prevalence of bacteriuria is unknown.

In this study we studied bacteriuria, significant macroscopic pathological changes and histological cystitis in culled sows at slaughter since this provided an opportunity to obtain non-contaminated urine samples by direct aspiration from the bladder. Since only one urine sample contained a mixed culture, the employed method was found to be very efficient in avoiding contamination. The low number of urine samples with a mixed culture is in contrast to a previous study that reported a higher level of mixed infections [25]. Our study only included sows with bladders containing urine. It can produce a systematic error in the observed prevalence since sows with cystitis may urinate more frequently and thereby have a greater risk of being slaughtered with an empty bladder. In our recent study (data not shown) however, no such difference between empty and full bladders could be established.

Approximately one third of the sows sampled in the study were shown to have bacteriuria. Similar prevalence of bacteriuria has been reported in a previous study where urine was collected by cystocentesis (27%) [13] as well as in other studies where samples were collected from the bladder mucosa (23–38%) [17, 19, 20]. In the study with the 27% prevalence the cut-off for a positive urine culture was ≥ 10^5^. If we use the same cut-off in our study the prevalence would be 30% (53/176). A one-tailed calculation for the significance of the difference between two independent proportions, comparing the 27% and 30% prevalence, gives a p-value of 0.05, meaning that the difference is borderline significant.

The last survey on bacteriuria prevalence in Denmark was conducted in 1994 and showed a prevalence of 19% in live sows from production herds [11]. This is lower than the prevalence found in this study, but it could be expected as the sows from that study were still reproductively active and the sows in our study could have been culled for reasons related to UTI.

Different studies have found very different results when comparing the prevalence of macroscopic pathological changes. It can be explained by the different definitions of significant changes and which changes should be present for the urinary bladder to be classified as abnormal. For example one study [13] registered congestion and bladder stones, another defined cystitis by congestion, thickening of the wall, presence of edema or presence of pus without reporting the prevalence of each specific finding [20]. A third study reported presence of low, moderate and high level of cystitis, without clearly specifying what pathological changes it was based on [15].

When evaluating the bladders macroscopically mucosal redness was strongly associated with presence of pus, concrements and a higher bladder weight. In all cases except for presence of concrements it was the intense redness that was the cause of the significance. When observing concrements in the bladder an association with mild redness was found. It suggests that the presence of mild redness may be a sign of a mechanical irritation from the concrements whereas the intense redness could indicate presence of an actual inflammation in the mucosa. This hypothesis is supported by the fact that mild redness was not associated with presence of hyperemia in the histological examination. Attention should therefore be given to distinguish between mild and intense redness when evaluating bladder mucosa macroscopically.

Furthermore, presence of concrements does not indicate cystitis in itself, but should be held up against other findings indicative of inflammation [13, 20]. A Danish study from 1997 [21] did provide detailed information about pathological findings. Compared to results from our study, they found significant pathological changes in 29% of samples, which is very similar to the frequency observed in our study (27%). However, the frequencies of concrements (13%) and thickening of the bladder wall (4%) reported in this older Danish study [21] differed from those we observed (27% and 2%, respectively). The significant difference in the prevalence of concrements (P < 0.001) could be consequent to the fact that Danish sows today are loose-housed during pregnancy and therefore could have difficulties in reaching the drinking station, resulting in water deprivation and higher risk for developing concrements in the bladder [30].

A significantly higher weight of the bladders was seen in relation to presence of pus and intense redness. It could indicate that the weight of the bladder can be an objective measure for the presence of other pathological changes. However, in this study information about the parity of the sows was unavailable, so correction for this was not included. Even though information of the parity of the sow is not included in this study, an older Danish study found that the weight of the bladder doubled if changes indicative of cystitis were present, after correcting for parity [31].

The prevalence of histopathological findings, indicating cystitis, in our study was 46%, which differs significantly from only one previous study which found a prevalence of 27% [20]. It could be explained by different cut-offs as our study defined neutrophilic infiltration as 1 cell/HPF and in the other study it was 5 cells/HPF. Two other studies found prevalence of 41% [16] and 53% [13] respectively, which is not significantly different from the prevalence found in our study. The first study (prevalence 41%) included mainly bladders with macroscopic signs of inflammation and could thereby overestimate the prevalence among slaughtered sows. The second study, with 53% prevalence, used almost the same definitions as in our study, but they included lymphocytic foci as indicator of cystitis. Also, the study with 27% prevalence used lymphocytic foci as a cystitis diagnosis. In our study lymphocytic foci were not associated with any other histopathological signs of inflammation. Lymphocytic foci were therefore considered an occasional normal finding in the bladder of sows and massive infiltration of lymphocytes should be present for the sample to be considered as cystitis.

A very low number of neutrophils in each sample were seen, meaning that bladders with acute cellular inflammatory responses were rare in this study. Nevertheless, 12% of the samples showed chronic-active inflammation, indicating that cystitis is a recurrent illness in the sows.

The low number of samples with acute inflammation makes it difficult to discover any differences in the association between the stage of histopathological cystitis and bacteriuria and significant macroscopic pathological changes.

While it makes perfectly sense that bacteria cause an acute—or active—response, we would not necessarily expect to find a significant amount of bacteria in a bladder displaying purely chronic lesions. However, the bacteria colonize the mucosa, leading to bacteriuria in chronic cystitis. Also the chronicity of the cystitis may be due to complicating factors (e.g. anatomical) that facilitate continuous access of bacteria to the urinary tract.

This study shows that 69% of the samples with bacteriuria harbored *E. coli*. This result correlates well with other studies conducted the last 20 years [13, 17, 25, 26]. The fact that only 2 samples of *E. coli* had a CFU counts lower than 10^3^, implies that *E. coli* is a likely cause of cystitis in sows, as described both in humans and other animal species where UTI is a common disease condition [32–34].

As earlier studies disagree on how important it is for the individual sow to have cystitis, it can be discussed whether it is a problem that approximately 1/3 of the sows have signs of cystitis and/or bacteriuria. Further studies should examine the effect of having UTI on reproductive parameters in producing sows.

For investigating UTI in live sows, a useful point-of-care diagnostic test should be chosen. For a simply research-related purpose, principally every test at any cost could be used, but for use at the farm as a diagnostic tool, the price and inconvenience of the test should be considered. As a start one could seek inspiration in the human and small animal veterinary fields. Further studies could make diagnostic evaluation of different tests using the cut-off for histopathological cystitis of > 10^3^ CFU as gold standard.

## Conclusion

The prevalence of cystitis in culled sows investigated in this study was 34% based on bacteriological examination, 27% based on macroscopic examination and 46% based on histological examination.

These results show that chronic cystitis can be difficult to observe macroscopically and most of the acute signs seen macroscopically are related to chronic active lesions seen microscopically.

Significant associations were found between the three indicators of cystitis: bacteriuria, pathological and histological lesions of the bladder. The cut-off for bacteriuria of ≥ 10^3^ CFU/mL seems to be appropriate for evaluating urine cultures obtained by cystocentesis in culled sows. As to macroscopic examination of bladders, concrements and mild redness should not be indicative of cystitis on their own.

## Methods

### Study design and sample size

A cross-sectional prevalence study was conducted. In total 179 sows were slaughtered at a Danish slaughterhouse on eight different days between March and September 2014. This sample size was considered acceptable since it provided a maximum allowable error (L) of 0.07 based on the prevalence of bacteriuria, macroscopical and histological lesions of the bladder observed in previous studies and in a pilot study (30% on average for all three indicators, data not shown). The pilot study was conducted by the main author on the same slaughterhouse as used in the main study. On each sampling day, 15–30 sows were sampled during four hours. The first sow from each herd was selected as it entered the slaughter line. Only sows with bladders containing urine were included in the study. Sows with visibly contaminated pelvic area were excluded. Later sows with missing histology registrations were excluded, leaving a final sample size of 176 sows.

### Herd data

The characteristics of the herds from which the sows originated were recorded according to geographical origin, sow inventory, production type and Specific Patogen Free (SPF) status. This information was obtained from the Danish Central Husbandry Register [36] and from the Danish SPF-Sus Herd Database [37].

### Bladder collection and urine sampling

The bladder was removed at the start of the slaughter line and the urethra was closed as close to the bladder as possible with a plastic strip to avoid contamination. 15–20 mL of urine were collected aseptically by cystocentesis using a 21G syringe within 5 h after removal of the bladder. The bladders were kept closed and stored at 10 °C (± 5 °C) until macroscopic evaluation 16–20 h later.

### Bacteriological examination

**Fig. 4:**
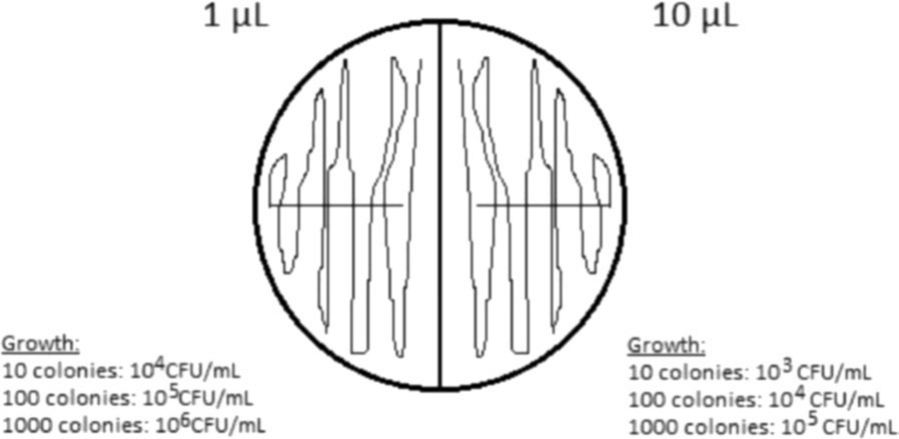
Interpretation chart for semi-quantitative colony forming unit counts of sow urine inoculated on blood agar. The urine samples were inoculated on standard 5% calf blood agar immediately after collection. Semi-quantitative colony counts were obtained by inoculating half an agar plate with 1 µL and the other half with 10 µL urine. Following aerobic incubation for 18–26 h at 37 °C, the number of colonies on each half of the agar plate were counted in order to determine the number of colony forming units per ml. If the two sides of the agar plate gave inconsistent results, the 10 µL side was used to maximize accuracy. The one µL loop determined the result for samples where doubt about the 10^5^ and 10^6^ categories occurred

The urine samples were inoculated on standard 5% calf blood agar immediately after collection. Semi-quantitative colony counts were obtained by inoculating half an agar plate with 1 µL and the other half with 10 µL urine. Following aerobic incubation for 18–26 h at 37 °C, the number of colonies on each half of the agar plate were counted according to Bjerrum et al. [38] in order to determine the number of colony forming units (CFU) per mL. The number of colonies on each side of the plate was estimated as described in Fig. 4. If the two sides of the agar plate gave inconsistent results, the 10 µL side was used to maximize accuracy, as previously recommended [39]. The 1 µL loop determined the result samples were doubt about the 10^5^ and 10^6^ categories occurred.

One representative colony type was sub-cultured from each plate and subjected to species identification using a MALDI-TOF mass spectrometer (VITEK® MS, bioMerieux, France).

Significant bacteriuria was defined by bacterial counts of 10^3^ CFU/mL or more [40]. Less than 10^3^ CFU/mL was considered as contamination, as previously recommended [39].

We did not identify *A. suis* since the aerobic culture conditions used in our study are not appropriate for isolation of anaerobic bacteria. As *A. suis* is a habitant of the preputium of boars and not naturally occurs in the urogenital tract of sows, it is unlikely a major cause of UTI in sows that are inseminated artificially [27, 35].

### Macroscopic bladder pathology

Each bladder was opened in the dorsal midline from the urethra to the apex with clean scissors. The urine was evaluated and categorized as normal urine or with presence of pus, blood and/or concrements. Presence of mucosal redness and thickening of the bladder wall was recorded. Mucosal redness was categorized as intense redness (dark pink to red) or mild redness (light or pale pink). Thickening of the bladder wall was categorized as normal or thick and was evaluated by palpation and stretching of the tissue. The bladder was weighed after removal and recording of any content using a standard digital scale with a precision of 1 g. Macroscopical lesions were defined as either acute or chronic; acute lesions included intense redness of the mucosa or concomitant presence of pus and mild redness; lesions were categorized as chronic in presence of thickening of the bladder wall.

### Histopathology

Tissue samples (2 × 3 cm of the bladder wall) were obtained from the middle of the bladder, in both the length and the width of the body of the bladder. All tissue samples were fixed in 10% formalin, processed for histopathology, and stained with hematoxylin and eosin. Lymphocytic foci, edema and hyperemia were evaluated at 200× magnification (20× objective). For cell count, 400× magnification (40× objective) and a net ocular with a grid size of 0.5 × 0.5 mm was used. Mononuclear cells and neutrophils in the submucosa were counted in three fields of view from each tissue sample. The three fields of view were in the middle of the sample and half-way from the sample edge on each side of the middle. Total cell count of mononuclear cells and neutrophils was calculated as the average of the three counts. Acute cystitis was defined by histopathology as presence of neutrophils in combination with edema and/or hyperemia. Chronic cystitis lesions were defined as presence of more than 40 mononuclear cells/HPF without other signs of inflammation. HPF was defined as one field of view in the microscope at 40× magnification and was chosen as the magnification at which the cells could be distinguished, and a substantial amount of the tissue piece was observable. If more than 40 mononuclear cells /HPF were present in combination with the lesions of acute cystitis the lesions were defined as chronic-active [13, 41].

### Statistics

Variables and categories used for prevalence calculations and statistics are shown in Table 5. All statistical analyses were performed using the free statistical software package “R”, version 3.1.1 [42]. Simple prevalence calculations and frequency distributions were determined as summary statistics. Test for normality was performed with the qqnorm function and the Shapiro–Wilk test.

**Table 5:** Variables recorded in bacteriology (urine samples), macroscopic pathological evaluation (bladders) and histopathological evaluation (bladders)

Variable	Categories
Bacteriology	
Number of colony forming units pr. mL (CFU/mL)	0, < 10^3^, 10^3^, 10^4^, 10^5^, 10^6^
Macroscopic organ evaluation	
Weight	Weight in grams (1 g accuracy)
Content	Normal urine, presence of pus, blood and/or concrements
Hyperemic mucosa	No redness, mild redness (light or pale pink), intense redness (dark pink to red)
Thickening of the bladder wall	Present/not present
Histopathology	
Lymphocytic foci	Foci/no foci
Edema	Edema/no edema
Hyperemia	Hyperemia/no hyperemia
Number of mononuclear cells	0–?
Number of neutrophils	0–?
Acute inflammation	Neutrophils in combination with edema and/or hyperemia
Chronic inflammation	> 40 mononuclear cells/HPF*, no other inflammatory signs
Chronic-active inflammation	> 40 mononuclear cells /HPF* in combination with the lesions of acute cystitis

*HPL High power field

Significance level was set to p ≤ 0.05. Chi square and Fisher’s exact tests were used for testing associations between categorical variables recorded by bacteriology, macroscopic pathology and histopathology. Students t-test, Kruskal–Wallis and one-way ANOVA tests were used for continuous variables depending on whether the data was normally distributed. The one-way ANOVA test was followed by a Tukeys HSD test.

## Data Availability

The datasets used and/or analysed during the current study are available from the corresponding author on reasonable request.

## Abbreviations

CFU: Colony forming units
HPF: High power field
OR: Odds ratio
SPF: Specific Pathogen Free
Spp.: Species (*pluralis*)
UTI: Urinary tract infection

## References

[1] Isling LK, Aalbaek B, Schroder M, Leifsson PS (2010). Pyelonephritis in slaughter pigs and sows: morphological characterization and aspects of pathogenesis and aetiology. Acta Vet Scand.

[2] Carr J (1991). Urocystitis and pyelonephritis in the sow. Pig Vet J.

[3] Petersen B (1983). Methods of early recognition of puerperal and fertility disorders in the sow. Livest Prod Sci.

[4] D'Allaire S, Drolet R, Chagnon M (1991). The causes of sow mortality: a retrospective study. Can Vet J.

[5] Chagnon M, D'Allaire S, Drolet R (1991). A prospective study of sow mortality in breeding herds. Can J Vet Res.

[6] Lopez R (2008). Low reproductive performance and high sow mortality in a pig breeding herd: a case study. Ir Vet J.

[7] Spillane P (1999). Urocystitis and endometritis in a 1,000 sow unit. Pig J.

[8] Both G, Moller K, Busse FW (1980). Relationships between reproductive disorders and urinary tract infections in pigs. I. Investigation of urine samples using bacteriological test strips. Tierarztl Umsch.

[9] Wegmann G. Bacteriological and pathological studies on slaughtered sows with and without reproductive disorders, with special reference to urinary tract infections. Thesis, Tierarztliche Fakultat, Ludwig-Maximilians-Universitat, Munchen, Germany. 1993.

[10] Christensen G, Vestergaard K. Slagtefund fra udvidet diagnostik (USK) på udsættersøer fra 10 sobesætninger. Meddelelse nr. 657 [Slaughter pathology on culled sows from 10 sow herds. Report 657]. SEGES Danish Pig Research Center. 2004. https://svineproduktion.dk/publikationer/kilder/lu_medd/2004/657. Accessed 23 April 2023.

[11] Thorup F. Betydningen af urinvejsinfektion for søernes reproduktion. Meddelelse nr. 271 [Importance of urinary tract infections for the sow reproduction. Report 271]. SEGES Danish Pig Research Center. 1994. https://svineproduktion.dk/publikationer/kilder/lu_medd/medd/271. Accessed 23 April 2023.

[12] Grahofer A, Björkman S, Peltoniemi O (2020). Diagnosis of endometritis and cystitis in sows: use of biomarkers. J Anim Sci.

[13] Bellino C, Gianella P, Grattarola C, Miniscalco B, Tursi M, Dondo A, D'Angelo A, Cagnasso A (2013). Urinary tract infections in sows in Italy: accuracy of urinalysis and urine culture against histological findings. Vet Rec.

[14] Carr J, Walton JR (1993). Bacterial flora of the urinary tract of pigs associated with urocystitis and pyelonephritis. Vet Rec.

[15] Schnurrbusch U, Ropke M, Lindner A (2009). Results of diagnostic examination of the genital organs of infertile sows in the years 2005–2007. Prakt Tierarzt.

[16] Christensen G, Vraa-Andersen L, Nielsen EO, Bille-Hansen V (1995). Diagnostik Af Urinblærebetændelse Hos Slagtesøer [Diagnostics of urocystitis in culled sows]. Dansk Veterinærtidsskrift.

[17] Kjelvik O, Karlberg K, Hofmo P (2002). Urinveislidelser hos purker med hovedvekt pa cystitter [Urinary tract infections in sows with emphasis on cystitis]. Norsk Veterinærtidsskrift.

[18] Moura R, Caldara FR, Foppa L, Machado SP, Nääs IA, Garcia RG, Gonçalves LMP (2018). Correlation between urinary tract infection and reproductive performance of sows. Braz J Vet Res Anim Sci.

[19] Thorup F. Antibiotikabehandling omkring løbning i besætninger med lav kuldstørrelse. Meddelelse nr. 9 [Antibiotic treatment at insemination in herds with few total born. Report 9]. SEGES Danis Pig Research Center. 1990. https://svineproduktion.dk/publikationer/kilder/lu_medd/medd/9. Accessed 23 April 2023.

[20] Biksi I, Takacs N, Vetesi F, Fodor L, Szenci O, Fenyo E (2002). Association between endometritis and urourocystitis in culled sows. Acta Vet Hung.

[21] Christensen G (1997). Udvidet slagtedyrsdiagnostik på udsættersøer (USK repro). 1. Normalforekomst af spontane forandringer i kæns- og urinvejsorganer, mavesæk og plucks [Extended slaughter pathology investigation (USK repro)-2. Correlations between culling causes, urogenital lesions, nutritional status and season]. Dansk Veterinærtidsskrift J..

[22] Monteiro MS, Matias DN, Poor AP, Dutra MC, Moreno LZ, Parra BM (2022). Causes of sow mortality and risks to post-mortem findings in a Brazilian intensive swine production system. Animals.

[23] Cernat M, Skampardonis V, Papadopoulos GA, Kroustallas F, Chalvatzi S, Petridou E (2021). Urinary tract infections in culled sows from Greek herds: prevalence and associations between findings of histopathology, bacteriology and urinalysis. Porcine Health Manag.

[24] Björkman S, Kauffold J, Kaiser MØ (2023). Reproductive health of the sow during puerperium. Mol Reprod Dev.

[25] Sipos W, Grahofer A, Fischer L, Entenfellner F, Sipos S (2014). Keimspektrum des Urogenitaltraktes von Sauen mit Fertilitätsstörungen. WTM.

[26] Mazutti K, Locatelli Dittrich R, Lunardon I, Kuchiishi SS, Lara AC, Zotti E, Alberton GC (2013). Evaluation of the reagent test strips and microscopic examination of urine in the diagnosis of urinary tract infection in sows. Pesq Vet Bras.

[27] Jones JET. Urocystitis and pyelonephritis in sows caused by *Corynebacterium suis*: epidemiological considerations. In: Proceedings of the society for veterinary epidemiology and preventive medicine. 1987;128–132.

[28] Walker RL, MacLachlan NJ (1989). Isolation of *Eubacterium suis* from sows with urocystitis. J Am Vet Med Assoc.

[29] Liebhold M, Wendt M, Kaup FJ, Drommer W (1995). Clinical, and light and electron microscopical findings in sows with urocystitis. Vet Rec.

[30] Maes DGD, Vrielinck J, Millet S, Janssens GPJ, Deprez P (2004). Urolithiasis in finishing pigs. Vet J.

[31] Christensen G (1997). Udvidet slagtedyrsdiagnostik på udsættersøer (USK repro). 1. Normalforekomst af spontane forandringer i kæns- og urinvejsorganer, mavesæk og plucks [Extended slaughter pathology investigation (USK repro)-1. Prevalence of spontaneous lesions in the urogenital tract, stomach and plucks]. Dansk Veterinærtidsskrift..

[32] Marques C, Gama LT, Belas A, Bergstrom K, Beurlet S, Briend-Marchal A (2016). European multicenter study on antimicrobial resistance in bacteria isolated from companion animal urinary tract infections. BMC Vet Res.

[33] McMeekin CH, Hill KE, Gibson IR, Bridges JP, Benschop J (2017). Antimicrobial resistance patterns of bacteria isolated from canine urinary samples submitted to a New Zealand veterinary diagnostic laboratory between 2005–2012. N Zeal Vet J.

[34] McLellan LK, Hunstad DA (2016). Urinary tract infection: pathogenesis and outlook. Trends Mol Med.

[35] Jones JET (1984). Urocystitis and pyelonephritis associated with *Corynebacterium suis* infection in sows. Vet Annu.

[36] Danish Husbandry Register (CHR). Danish Veterinary and Food Administration. 2017. https://chr.fvst.dk/chri/faces/frontpage?_adf.ctrl-state=15tki6lee3_7. Accessed 17 May 2017.

[37] The Health Status Management Database (Sundhedsstyringen). SEGES. 2017. http://spfsus.dk/. Accessed 17 May 2017.

[38] Bjerrum L, Grinsted P, Højbjerg T (2012). Diagnostik af urinvejsinfektion i almen praksis [Diagnostic of cystitis in general practice]. Maanedsskrift for Praktisk Laegegerning.

[39] Frimodt-Møller N, Espersen F (2000). Evaluation of calibrated 1 and 10 µl loops and dipslide as compared to pipettes for detection of low count bacteriuria in vitro. APMIS.

[40] Weese JS, Blondeau JM, Boothe D, Breitschwerdt EB, Guardabassi L, Hillier A (2011). Antimicrobial use guidelines for treatment of urinary tract disease in dogs and cats: antimicrobial guidelines working group of the international society for companion animal infectious diseases. Vet Med Int.

[41] Newman SJ, Confer AW, Panciera RJ, McGavin MD, Zachary JF (2007). Urinary system. Pathological basis of veterinary disease.

[42] RCoreTeam (2014). R: a language and environment for statistical computing.

